# Spirometry to manage asthma in children: study protocol for a randomised controlled trial (SPIROMAC)

**DOI:** 10.1186/s13063-025-09104-1

**Published:** 2025-09-29

**Authors:** Victoria Bell, Nicole Sergenson, Seonaidh Cotton, Chukwuemeka David Emele, Ruth Thomas, Lorna Aucott, Mark Forrest, Erol A. Gaillard, Erika J. Kennington, Graeme MacLennan, Ian Sinha, Thenmalar Vadiveloo, Neil W. Scott, Steve Turner

**Affiliations:** 1 Centre for Healthcare Randomised Trials, University of Aberdeen, Aberdeen, UK; 2 Aberdeen Centre for Evaluation, University of Aberdeen, Aberdeen, UK; 3 Department of Respiratory Sciences, NIHR Biomedical Research Centre (Respiratory Theme), University of Leicester, Leicester, UK; 4 Asthma + Lung UK, London, UK; 5 Respiratory Unit, Alder Hey Children’s Hospital, Liverpool, UK; 6 Biostatistics and Health Data Science, University of Aberdeen, Aberdeen, UK; 7 Royal Aberdeen Children’s Hospital, NHS Grampian, Aberdeen, UK

**Keywords:** Asthma, Child, Randomised controlled trial, Spirometry

## Abstract

**Background:**

Asthma affects over 1 million children across the UK, and preventative treatment is guided subjectively by patient symptoms. Spirometry is an objective test of lung function and can be used in children to guide treatment. However, current guidelines do not indicate how asthma treatment should change in the context of changing spirometry results. This study will evaluate how spirometry can be used to guide asthma treatment and reduce the risk for asthma attacks in children.

**Methods:**

This is a multi-centre, randomised controlled trial. Children aged 6–15 years, who have a diagnosis of asthma and have had an exacerbation requiring oral or intravenous corticosteroids in the previous 12 months, will be eligible. Exclusion criteria include being unable to provide spirometry measurement at baseline assessment, having another chronic respiratory condition and being currently treated with maintenance oral steroids or biologicals. Participants will be recruited in both primary and secondary care settings and will be randomised to either receive asthma treatment guided by spirometry plus symptoms (intervention group) or asthma treatment guided by symptoms only (standard care group). Within the spirometry group, treatment recommendations will be dependent on changes in spirometry measurements. Participants will attend assessments 3, 6, 9 and 12 months post-randomisation, where treatment recommendations will be made. The primary outcome is the number of asthma attacks per participant requiring treatment with 1–7 days of oral or intravenous corticosteroid over 12 months, as recorded by the participant or parent. Secondary outcomes include time to first attack, any asthma attack, adverse events, dose of inhaled corticosteroids, asthma control and quality of life. Adherence to inhaled corticosteroid treatment is measured by an electronic logging device.

**Discussion:**

This study will evaluate whether asthma treatment guided by spirometry will reduce future asthma attacks in children. Our findings may be relevant to national and international asthma guidelines.

**Trial registration:**

ISRCTN, ISRCTN31849868. Registered on 01.07.2022. Prospectively registered.

**Supplementary Information:**

The online version contains supplementary material available at 10.1186/s13063-025-09104-1.

## Administrative information

Note: the numbers in curly brackets in this protocol refer to SPIRIT checklist item numbers. The order of the items has been modified to group similar items (see http://www.equator-network.org/reporting-guidelines/spirit-2013-statement-defining-standard-protocol-items-for-clinical-trials/).

**Table:** 

Title {1}	Spirometry to manage asthma in children: study protocol for a randomised controlled trial (SPIROMAC)
Trial registration {2a and 2b}.	ISRCTN, ISRCTN31849868. Registered on 01.07.2022. Prospectively registered.
Protocol version {3}	Version 8.0, 29.04.2025.
Funding {4}	National Institute for Health Research (NIHR) Efficacy and Mechanisms Evaluation (EME), project number NIHR129819
Author details {5a}	^1^ Centre for Healthcare Randomised Trials, University of Aberdeen, Aberdeen, UK ^2^ Aberdeen Centre for Evaluation, University of Aberdeen, Aberdeen, UK ^3^ Department of Respiratory Sciences, NIHR Biomedical Research Centre (Respiratory theme), University of Leicester, Leicester, UK ^4^ Asthma + Lung UK, London, UK ^5^ Respiratory Unit, Alder Hey Children's Hospital, Liverpool, UK ^6^ Biostatistics and Health Data Science, University of Aberdeen, Aberdeen, UK ^7^ Royal Aberdeen Children's Hospital, NHS Grampian, Aberdeen, UK
Name and contact information for the trial sponsor {5b}	Co-sponsor 1. University of Aberdeen, Level 1, Health Sciences Building, Foresterhill, Aberdeen, AB25 2ZB, researchgovernance@abdn.ac.uk Co-sponsor 2. NHS Grampian, Level 1, Health Sciences Building, Foresterhill, Aberdeen, AB25 2ZB, researchgovernance@abdn.ac.uk
Role of sponsor {5c}	The sponsor played no part in study design; and will play no part in the collection, management, analysis and interpretation of data; writing of the report; and the decision to submit the report for publication.

## Introduction

### Background and rationale {6a}

Each year, 20% of the 1.1 million children with asthma in the UK has an asthma attack [1]. Many attacks can be prevented by regular routine assessments and appropriate asthma preventer treatment; however, current decision-making around preventer treatment is subjective and guided by current symptoms. There is a need for a validated objective test to guide preventer treatment. Spirometry is a reproducible test of lung function [2] and can be obtained in > 94% of school-aged children [3].

Currently, there is weak or no evidence underpinning those guidelines which recommend spirometry should be used as part of asthma management. One of five current asthma guidelines [4] cite a single observational study [5] to support the role of spirometry in monitoring asthma. Three guidelines, including one which was until very recently valid for the UK [6–8], cite no supporting evidence but recommend that spirometry should be done. One UK guideline [9] does not recommend regular spirometry in children, and this is consistent with the recommendation of the new, single 2024 guideline [10]. Two guidelines suggest that treatment could be changed when FEV_1_ is < 80% of predicted values [6, 7]. No guidelines give clinically meaningful guidance advising clinicians how or when to amend asthma treatment when considering spirometry results.

Forced Expiratory Volume in one second (FEV_1_) is the preferred spirometric index in asthma clinical trials [11]. Associations have been made between a “low” value of FEV_1_ on a single occasion and increased risk for future asthma attacks [5, 12–15]. The period of follow-up after the FEV_1_ measurement were 3 months [12], 12 months [5, 13], 3 years [14] and 4 years [15]. An additional paper [16] found that FEV_1_ results are within the normal range (i.e. between 70 and 130% of predicted) in most children with asthma, and this means that using one-size-fits-all cut-off values to trigger change in treatment is unhelpful in identifying individuals at risk for attack; FEV_1_ < 80% is recommended in some guidelines [6, 7]. A further limitation of using one-size-fits-all cut offs is that some asymptomatic individuals have an FEV_1_ persistently below 80% (i.e. always “abnormal”) [6, 7].

It is now recognized that asthma is a heterogeneous condition [17], and a one-size-fits-all approach to asthma preventer treatment is not appropriate. An individualised approach to interpreting spirometry values may be more meaningful for the risk of future asthma attacks [4], and our observational study [12] describes how a 10% fall in FEV_1_ over a 3-month interval where children had good asthma control and FEV_1_ was within the “normal” range was associated with a 28% increased risk for an asthma attack over the next 3 months.

We have established that there is an element of equipoise across the UK in the application of spirometry to asthma monitoring [18]. There is a need for rigorous evaluation of how spirometry can be used to guide asthma treatment and reduce the risk for asthma attacks in children, and SPIROMAC is designed to achieve this.

SPIROMAC aims to contribute to an improvement in the health of the UK public by providing a method to reduce asthma attacks in children and also describes how the methodology works.

### Objectives {7}

The overarching aim of the trial is to compare treatment guided by spirometry plus symptoms against treatment guided by symptoms alone (standard care) in children with asthma who are at risk of an asthma attack. There are efficacy and mechanism aims:

(i) Efficacy: To determine the efficacy of treatment guided by spirometry plus symptoms compared to treatment guided by symptoms alone in children with respect to: asthma attacks up to 12 months post randomisation; and asthma control (symptoms); health-related quality of life; adverse events and dose of inhaled corticosteroids.
(ii) Mechanism: To determine whether treatment guided by spirometry plus symptoms (compared to treatment guided by symptoms only) leads to improved air flow (FEV_1_), lung volume (FVC) and reduced eosinophilic airway inflammation (fractional exhaled nitric oxide, FeNO) and this physiological change is associated with reduced attacks.

We hypothesise that asthma treatment guided by spirometry will improve expiratory flows and volumes and reduce FeNO, and that this will lead to reduced asthma attacks. SPIROMAC will therefore determine whether the spirometry-guided treatment reduces attacks by achieving one or more of the following physiological changes:

- Increased airway flow (FEV_1_)
- Increased lung volume (forced vital capacity, FVC)
- Reduced eosinophilic airway inflammation, as evidenced by FeNO [19].

### Trial design {8}

A multicentered randomised controlled trial will compare asthma treatment guided by spirometry plus symptoms compared to treatment guided by symptoms alone. We will measure spirometry in all participants, and FEV_1_ will be used within an algorithm to recommend treatment in the intervention arm. This recommendation will be reviewed by the Local clinical team who will determine the final treatment. All participants, carers and usual clinical teams will be blinded to spirometry results unless there is clinical need. A 9-month internal pilot phase was included, and its objectives were to assess recruitment, equipoise, feasibility and acceptability of the algorithm and quality of data collected. The expectation was that at the end of the pilot there would be at least 18 sites open for recruitment and 48 participants recruited. The objectives of the pilot were met; 32 sites and 96 children were recruited and this informed progression to full trial without any modifications to trial processes. Data collected during the pilot phase will be included in the SPIROMAC analysis.

## Methods: participants, interventions and outcomes

### Study setting {9}

Children will be recruited from secondary care centres across the UK and in primary care centres in the East of England. A list of participating study centres can be found on the SPIROMAC study website [20].

### Eligibility criteria {10}

The inclusion criteria for participants are:

- Aged 6–15 years
- Asthma diagnosis confirmed by a doctor or a respiratory/asthma specialist nurse (or Read code for asthma if recruited in primary care)
- Patient/parent reported-asthma attack treated with oral and/or intravenous corticosteroids in the 12 months prior to identification for the study
- Be able to perform spirometry
- Be able to understand written/spoken English.

The exclusion criteria for participants are***:***

- Not being able to perform spirometry satisfactorily (*Being unable to provide spirometry included providing spirometry but not meeting the quality control criteria for that unit or failing to provide any spirometry. To reduce the incidence of this children will be advised how to perform spirometry assessment by an experienced clinician. When adequate spirometry cannot be obtained after several efforts, the child will be given the opportunity to practice at home and be reassessed for eligibility at a later date.*)
- Presence of another chronic respiratory condition
- Current treatment with maintenance oral steroids or biologicals

### Who will take informed consent? {26a}

Consent is taken by researchers trained in Good Clinical Practice (GCP). Written consent is obtained from parent(s)/carer(s) and (where appropriate) from the child. If the child does not provide written consent, they are asked to give verbal assent. Children who turn 16 during follow-up are given the opportunity to provide written re-consent.

### Additional consent provisions for collection and use of participant data and biological specimens {26b}

Families are asked if they wish to consent to provide saliva for DNA extraction for genes that may be associated with asthma and allergy. This is optional.

## Interventions

### Explanation for the choice of comparators {6b}

Justification for this is given in the “Background and rationale” section.

### Intervention description {11a}

The intervention groups are as follows:

- Intervention: treatment guided by spirometry plus asthma control (symptoms)
- Standard care: treatment guided by asthma control (symptoms)

In the intervention arm, treatment is guided by asthma control, adherence to preventer treatment, recent asthma attacks, recent increase in treatment, short-acting beta-2 agonists (SABA) use, current treatment (ICS dose) plus spirometry (FEV_1_).

In the standard care arm, treatment uses the same factors but does not include spirometry (FEV_1_).

These data, shown in Table 1, are entered into the algorithm which is embedded into the study website and a treatment recommendation is made. This recommendation will be reviewed by the local clinical team who will determine the final treatment.

**Table 1:** Data applied each visit for algorithm in intervention and standard care arms

	**Intervention**		**Standard care**	
	**Baseline**	**Follow-up** **(3, 6, 9, 12 months)**	**Baseline**	**Follow-up** **(3, 6, 9, 12 months))**
Asthma Control (ACT/CACT; three categories)	✓	✓	✓	✓
Adherence (at baseline from parent report, at follow-up using electronic logging device if available)	✓	✓	✓	✓
Asthma attack in last 3 months (at baseline in last 6 months)	✓	✓	✓	✓
Step up in treatment since last study visit (at baseline in last 3 months)	✓	✓	✓	✓
SABA use	✓	✓	✓	✓
Current treatment (ICS dose)	✓	✓	✓	✓
FEV_1_	✓	✓		

The algorithm uses web-based software to apply a decision tree which is described in Additional File 1 and the treatment step table in accordance with national guidelines [9] shown in Additional File 2. Based on the factors shown in Table 1, the algorithm makes a recommendation as to whether there should be a step up or step down to a specified treatment or there should be no change to treatment. In some scenarios, e.g., if a child is already on the highest level of treatment, is consistently poorly adherent, or is on a treatment which is not specified in the treatment step table (Additional File 2) then the algorithm will suggest referral for a clinical opinion (RCO). The treatment step table (Additional File 2) then recommends what preventor medication the participant would step up or step down to for the next 3 months. The local clinical team and family will review the treatment recommendation and decide on the final treatment.

### Criteria for discontinuing or modifying allocated interventions {11b}

The local clinical team will review the treatment recommendation at each visit and decide whether to follow this recommendation based on their clinical judgement. The family can also request modification to the treatment at any visit. The reason for any modification will be documented. Participants can request not to have treatment recommendations suggested by the algorithm should they wish to. Between study visits, asthma treatment may be changed by another health care professional.

### Strategies to improve adherence to interventions {11c}

Adherence by local research teams to the interventions is facilitated by the web-based software which presents the researcher clear instructions as to what change in treatment (if any) is suggested. When the participants return for their next assessment, the algorithm will analyse the data that is entered to detect whether treatment has changed between assessments, e.g. changed by the participant’s GP. Our previous experience with a similar algorithm is that there is intentional non-adherence to approximately 20% of treatment recommendations [21, 22]. Adherence to ICS treatment by participants is facilitated by electronic logging devices, where there is a device compatible for the participant’s inhaler type.

### Relevant concomitant care permitted or prohibited during the trial {11d}

Usual care for participants continues throughout the trial. No care is prohibited.

### Provisions for post-trial care {30}

Usual care will be provided post-trial within the National Health Service (NHS).

### Outcomes {12}

#### Primary outcome

The primary outcome is the number of asthma attacks per participant which require treatment with 1–7 days of oral corticosteroids (OCS) and/or intravenous corticosteroids up to 12 months post randomisation reported by the participant or parent/carer. There is a 2019 UK national guideline which standardises OCS prescribing for an asthma attack in children [9]. For participants lost to follow-up, the primary outcome will be determined from primary and secondary care records by research staff.

#### Secondary outcomes

- Any asthma attack up to 12 months post randomisation (yes/no)
- Time to first asthma attack (days)
- Asthma control (participant reported asthma symptoms, using Asthma Control Test (ACT) or Children’s Asthma Control Test (CACT) as appropriate)
- Quality of life measured using the Paediatric Asthma Quality of Life Questionnaire (PAQLQ)
- Adverse events
- Dose of inhaled corticosteroids (ICS)

Additionally, spirometry measurements (FEV_1_, FVC) and FeNO during the 12-month follow-up will be included as outcomes in the mechanistic analysis.

For participants lost to follow-up, any asthma attack and time to first asthma attack outcome data will be collected from medical records.

### Participant timeline {13}

**Fig. 1:**
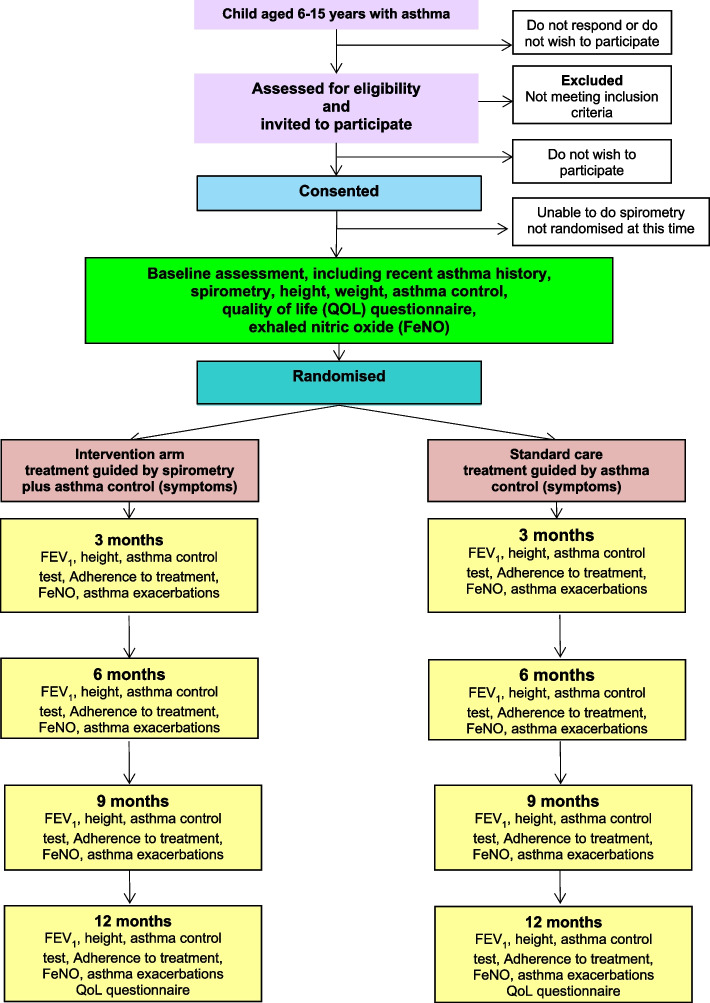
Participant’s timeline through the trial

Participants are invited to further assessments at 3, 6, 9 and 12 months after randomisation. There will be a 6-week window before and after each assessment date for that assessment appointment to take place. Figure 1 shows the participant’s journey through the SPIROMAC trial.

### Sample size {14}

The total sample size is 550. With 275 children in each arm, we will have 90% power (with 5% significance, 2-sided) to detect a 28% reduced risk for asthma attacks from 50% for participants in the control arm to 36% for the intervention arm, allowing for 5% of participants having incomplete primary outcome data.

### Recruitment {15}

In secondary care settings, eligible individuals are identified by their usual clinical team and approached in person or are sent a letter of invitation, which is accompanied by parent and child patient information leaflets (PIL). Those interested in taking part in the research can contact the research team and arrange an in-person appointment, where eligibility is confirmed, consent taken, baseline details collected and randomisation performed.

Within primary care research centres and primary care Participant Identification Centres (PIC), NRS Primary Care Network staff, CRN staff and practice staff identify eligible participants from practice records, and an invitation letter is sent accompanied by parent and child patient information leaflets (PIL). As above, those interested in taking part contact the research team or return the reply slip in a pre-paid envelope. Assessments of children identified in primary care take place in the primary or secondary care setting, depending on local resources.

## Assignment of interventions: allocation

### Sequence generation {16a}

After the participant gives their consent to take part in the trial, they are randomly allocated to either the intervention or standard care group using a computerised minimisation algorithm. Minimisation variables are centre, age (< 12 years, ≥ 12 years), sex and asthma severity according to British Thoracic Society/Scottish Intercollegiate Guidelines Network [BTS/SIGN] [9] treatment step (2, 3, 4, 5 or other, i.e. for children on a non-standard treatment regimen that cannot be immediately classified into a BTS step) including a random element (20%). The primary care research centres in East of England that undertake assessments in primary care will be collectively considered as one centre for randomisation.

### Concealment mechanism {16b}

The central, remote web-based randomisation system (using the minimisation algorithm which includes a random element) hosted by CHaRT (Centre for Healthcare and Randomised Trials) ensures allocation concealment.

### Implementation {16c}

The web-based randomisation system generates the allocation sequence. Trained research staff at each of the recruitment centres consent participants, collect their baseline assessments, then log into the central, remote web-based randomisation system. The web-based randomisation system assigns the participant to the intervention or control (standard care) arm.

## Assignment of interventions: blinding

### Who will be blinded {17a}

All research and clinical staff at recruiting centres, the participants and their families will be blinded to the randomisation allocation.

Spirometry data will be collected from the participants in both arms and entered into the study website. In participants randomised to the intervention arm, the web-based algorithm considers the spirometry results in the treatment recommendations. In participants randomised to the control arm, the algorithm will not consider the spirometry results.

### Procedure for unblinding if needed {17b}

There is no requirement for emergency unblinding procedures. This is because knowledge of whether a participant is in the control or intervention group will not impact on any management decisions being taken if an adverse event occurs.

## Data collection and management

### Plans for assessment and collection of outcomes {18a}

Data are collected by Local clinical teams at face-to-face appointments with participants. When a face-to-face visit is not possible, data will be collected via telephone appointment or postal questionnaire. Data is recorded on the study website via electronic case report forms and participant questionnaires at baseline and at 3, 6, 9 and 12 months (Table 2).

**Table 2:** Timing of outcome measures

	**Baseline assessment**	**Three-month assessment**	**Six-month assessment**	**Nine-month assessment**	**Twelve-month assessment**
Baseline characteristics, including recent asthma history	✓				
Spirometry	✓	✓	✓	✓	✓
Height	✓	✓	✓	✓	✓
Weight	✓				
Asthma Control Test	✓	✓	✓	✓	✓
Paediatric Asthma Quality of Life Questionnaire	✓				✓
Primary outcome (asthma attack)		✓	✓	✓	✓
Adherence to ICS treatment (from electronic logging device)		✓	✓	✓	✓
Exhaled nitric oxide (FeNO)	✓	✓	✓	✓	✓

Spirometry will be measured by staff with an Association of Respiratory Technology and Physiology (ARTP) qualification, or who have received in-house training or undertaken this as part of their routine NHS role. Spirometry will be standardised according to the Global Lung Initiative [23]. A clinically significant reduction in spirometry will be defined as FEV_1_/FVC below the lower limit of normal at the baseline assessment. At the following assessments, as a change of ± 1.6 in change score [24, 25] from the previous assessment.

The ACT [26] (for those aged 12 years and over) and CACT (for younger children) are validated questionnaires used in routine clinical practice to objectively determine asthma control. We will categorise the ACT or CACT score into the three “control states” (fully controlled, partly controlled or uncontrolled.) We will use the validated PAQLQ [27] to measure asthma quality of life at baseline and 12 months post randomisation.

We will measure FeNO in accordance with the standard methodology [28] using the NIOX Vero ® device (Circassia, Oxford, UK). The FeNO measurements will not contribute to the algorithm but are key to the mechanistic aspect of the study.

Participants can opt into providing a saliva sample for the mechanistic component of the trial. A sample can be collected at any trial visit via Oragene self-collection kits (DNA Genotek™, Ottawa, ON, Canada).

### Plans to promote participant retention and complete follow-up {18b}

There is a 6-week visit window around each of the follow-up assessments. Where possible, study follow-up visits are timed to coincide with routine clinic appointments. Follow-up appointments can be conducted by telephone or outcome data can be collected by postal questionnaire when a face-to-face visit is not possible. At 12 months, primary outcome data can be collected from the GP practice if the above methods are unsuccessful. Using this approach in a similar trial, we obtained primary outcome data in 99% of participants [21].

### Data management {19}

Data are stored on a secure server at the University of Aberdeen. All data are entered by centre staff or core trial team staff onto restricted access web-based case report forms and questionnaires. The web-based case report form includes integrated validation checks, such as range checks for data values to minimise data errors. To promote data quality, the core trial team staff perform quality control checks on the data to ensure that data entry is accurate and any missing data or data inconsistencies are addressed as soon as possible. Details of the data management procedures can be found within the Trial Monitoring Plan.

### Confidentiality {27}

Data are stored in accordance with GCP and comply with the requirements of the UK General Data Protection Regulations (GDPR) and the Data Protection Act 2018.

### Plans for collection, laboratory evaluation and storage of biological specimens for genetic or molecular analysis in this trial/future use {33}

Saliva samples are collected in Oragene collection kits (DNA Genotek™, Ottawa, ON, Canada). DNA will be extracted—the primary variant of interest is the Arg16Gly single nucleotide polymorphism of the gene coding for the Beta 2 adrenoreceptor.

## Statistical methods

### Statistical methods for primary and secondary outcomes {20a}

#### Efficacy analysis

Main analyses will be based on the Intention-to-Treat (ITT) principle. The primary outcome will be analysed using a negative binomial regression model that includes time in the study as the exposure (allowing inclusion of data from participants without complete follow-up data) with adjustment for age, sex, socioeconomic status, asthma severity (as evidenced by BTS treatment step) and centre (random effect). Secondary outcomes will be analysed in a similar manner using generalised linear models and the appropriate link function. Comparison of PAQLQ at the final assessment (12 months) between treatment groups will be assessed using analysis of covariance, adjusting for the covariates listed above and baseline PAQLQ. The influence of any missing data on the robustness of the findings will be examined using sensitivity analyses incorporating multiple imputation or other relevant strategies under alternative assumptions.

#### Mediational analysis

Over the 12-month period we will collect the mechanistic outcomes (FEV_1_, FVC and FeNO) at baseline and then every 3 months. We will use causal mediation analysis to explore the indirect effects of treatment mediated by increases in spirometry as measured by FEV_1_, FVC or a reduction in eosinophilic airway inflammation as evidenced by FeNO. We will explore any mechanistic relationship by using time‐to‐first‐asthma‐attack as an outcome in a survival analysis and accounting for repeatedly measured FEV_1_, FVC and FeNO, which is most likely subject to time‐varying confounding, using the methods outlined in Vansteelandt et al. [29]*.*

Safety and other data will be adjudicated at least annually by our oversight committees. Analysis will be fully specified in a Statistical Analysis Plan.

### Interim analyses {21b}

There are no planned interim analyses.

### Methods for additional analyses (e.g. subgroup analyses) {20b}

We plan the following sub-group analyses: age, sex, socioeconomic status, asthma severity.

### Methods in analysis to handle protocol non-adherence and any statistical methods to handle missing data {20c}

Per-protocol analysis and, if appropriate, a complier-adjusted causal estimation (CACE) will be explored to evaluate the influence of compliance on the treatment effect for the primary outcome. Multiple imputation and imputing a range of values for missing data under missing not at random assumptions will be used to explore the robustness of treatment effect estimates.

### Plans to give access to the full protocol, participant level-data and statistical code {31c}

The full protocol is publicly available on NIHR funding and awards website [30]^.^ Non-identifiable participant-level data may be available on request to the Chief Investigator (CI), Professor Turner (s.w.turner@abdn.ac.uk).

## Oversight and monitoring

### Composition of the coordinating centre and trial steering committee {5d}

The core trial team based in the coordinating centre (CI, trial manager, data coordinator, programmer) meets weekly. A Project Management Group (PMG) and Trial Steering Committee (TSC) oversee the project. The PMG meets every 3 months and comprises the CI, grant holders (including clinical, methodological and statistical expertise) and the core trial team. The TSC meets every 12 months and comprises an independent chair, independent clinical and methodological expertise, PPI representation and the CI.

### Composition of the data monitoring committee, its role and reporting structure {21a}

The Data Monitoring Committee (DMC) meets approximately every 12 months. The DMC comprises an independent chair and independent members with clinical and methodological expertise. The DMC reports to the chair of the TSC.

### Adverse event reporting and harms {22}

Within SPIROMAC only adverse events (AEs) and serious adverse events (SAEs) relating to use of spirometry equipment, the NIOX VERO or other study assessments are recorded. An asthma exacerbation is an outcome for this study and is not an AE or SAE. Hospitalisations for planned treatments, elective or emergency treatment will not be considered as an AE or SAE.

Research staff within centres collect data on occurrence of AEs and SAEs at each study visit. Serious related and unexpected AEs are reported to the REC within 15 days of CI becoming aware. All related SAEs will be summarised and reported to the Ethics Committee, the Funder and the Trial Steering Committee in their regular progress reports.

### Frequency and plans for auditing trial conduct {23}

The trial is monitored to ensure that it is being conducted as per protocol, adhering to Research Governance, the principles of GCP, and all other appropriate regulations. The approach to and extent of monitoring is specified in the trial monitoring plan and is appropriate to the risk assessment of the trial. Investigators and their host institutions are required to permit trial-related monitoring and audits to take place by the Sponsor and/or regulatory representatives.

### Plans for communicating important protocol amendments to relevant parties (e.g. trial participants, ethical committees) {25}

Changes to the protocol require the trial office to seek permission from the funder, sponsor, PMG, TSC, REC and NHS R&D offices. The study website and trial registry are updated with any relevant protocol amendments.

### Dissemination plans {31a}

Once the main trial findings have been published, a plain English summary of the findings will be sent to all the families involved in the trial. Trial findings will also be disseminated to professionals involved in the trial, including GPs, PIs and staff at recruiting centres. Our results will be presented at an international respiratory conference, e.g. European Respiratory Society. A paper describing the results will be submitted to a high impact factor journal. We will also disseminate results to relevant patient and clinical interest groups.

## Discussion

Spirometry is used by clinicians and recommended in many guidelines for monitoring of asthma in children, but evidence supporting this use of spirometry is lacking. SPIROMAC will use spirometry plus asthma control (symptoms) to guide asthma preventer treatment with the aim of reducing asthma attacks. Our randomised controlled trial will minimise confounding factors associated with asthma attacks. The mechanistic component of the study explores the link between treatment decisions being informed by spirometry and future asthma attacks.

Practical issues which we anticipate are related to recruitment, retention and assessment methodology. Recruitment is a challenge for most clinical trials, including ours, and we will use a network of recruiting centres, many of which recently participated in a similar study [21]. We are aware of centres that will not be able to participate because their clinicians use spirometry as part of usual care and are not prepared to deliver care without spirometry [18]. Non-attendance at follow-up assessments is anticipated and presents a challenge since our intervention requires spirometry results. However, for participants randomised to the intervention who are not able to attend for spirometry but who provide other information needed to deliver the algorithm by telephone, the algorithm will make a recommendation without spirometry (as it does in the standard care arm).To ensure we collect the primary outcome in as many participants as possible, we will contact family doctors of participants who do not attend or provide these data for the final assessment; using this approach previously, we achieved outcome data for 99% of participants [21]. Our preliminary work has identified that spirometry is likely to be measured by a range of staff at recruitment centres, including respiratory physiologists, doctors, asthma nurses and research nurses. To standardise measurement, ensure quality and to reflect real-life clinical practice, within SPIROMAC, spirometry can be performed by staff with ARTP qualification and in accordance with the international guideline [31], or by staff who do not have ARTP qualification but who have either received in-house training or by staff who already undertake this activity as part of their routine NHS activity (or have previously done so).

The use of maintenance and reliever treatment (MART) is an increasingly used approach, using a single ICS/LABA combination inhaler as both preventer and reliever inhaler. This presents an additional challenge for the algorithm because there is no single standard approach to MART. To address this heterogeneity, children who used MART are identified as being on regular ICS/LABA combination treatment and also as being on MART.

Where a suitable device is available (and local NHS IT policies permit use) an electronic logging device can be used to monitor adherence to preventer treatment. We have previously documented some of the issues associated with use of these logging devices [21].

We have adopted a hybrid approach to training, whereby research staff at recruiting centres watch a series of videos about the study at a time convenient to them. Prior to opening a centre, we convene a synchronous session with key staff to answer any questions and consider the local implementation of the study. This format of training has been well received [32]. We also convene research team online meetings every 2 months to identify and communicate operational issues as they arise, troubleshoot any problems and give staff at recruiting centres the opportunity to share best practice. These meetings are followed up with a study newsletter.

The SPIROMAC trial will complete participant follow-up in 2026 and report on its findings in 2027.

## Trial status

Recruitment began in January 2023 and is planned to complete during 2025. The current protocol is version 8.0 (dated 29/04/2025).

## Supplementary Information

Additional file 1. SPIROMAC Algorithm decision trees. Algorithm decision trees on which treatment recommendations are based

Additional file 2. SPROMAC Algorithm recommended treatment step table. Algorithm treatment recommendations

## Data Availability

Data may be available for collaborators on request to the CI, Professor Turner. (s.w.turner@abdn.ac.uk).

## Abbreviations

ACT: Asthma Control Test
AE: Adverse event
ARTP: Association of Respiratory Technology and Physiology
BTS/SIGN: British Thoracic Society/Scottish Intercollegiate Guidelines Network
CACT: Children’s Asthma Control Test
CHaRT: Centre for Healthcare Randomised Trials
CI: Chief Investigator
DMC: Data Monitoring Committee
DNA: Deoxyribonucleic acid
EME: Efficacy and Mechanism Evaluation
FEF_25-75_: Forced expiratory flow at 25–75% of forced vital capacity
FeNO: Fractional exhaled nitric oxide
FEV_1_: Forced exhaled volume in 1 second
FVC: Forced vital capacity
GCP: Good Clinical Practice
GDPR: General Data Protection Regulations
GP: General Practitioner
ICS: Inhaled corticosteroids
ISRCTN: International Standard Randomised Controlled Trial Number
ITT: Intention-to-Treat
LABA: Long-acting beta agonist
MART: Maintenance and reliever treatment
NHS: National Health Service
NIHR: National Institute Health Research
OCS: Oral corticosteroids
PAQLQ: Paediatric Asthma Quality of Life Questionnaire
PI: Principal Investigator
PIC: Participant Identification Centre
PIL: Patient Information Leaflet
PMG: Project Management Group
PPIE: Patient and Public Involvement/Engagement
R&D: Research and Development
REC: Research Ethics Committee
SABA: Short-acting beta-agonist
SAE: Serious adverse event
TSC: Trial Steering Committee
