# Supplementary material for: Spirometry to manage asthma in children: study protocol for a randomised controlled trial (SPIROMAC)
Source: Trials. 2025 Sep 29;26:373. doi: 10.1186/s13063-025-09104-1 (PMC12482643; doi:10.1186/s13063-025-09104-1)
Supplement: Supplementary file 1 — Additional file 1. SPIROMAC Algorithm decision trees. Algorithm decision trees on which treatment recommendations are based [file 13063_2025_9104_MOESM1_ESM.docx]

**Additional File 1 –Algorithm decision trees on which treatment recommendations are based**

**Decision tree - recruitment visit**

| **FINAL NUMBER** | **R1** | **R2** | **R3** | **R4** | **R5** | **R6** | **R7** | **R8** | **R9** | **R10** | **R11** | **R12** | **R13** | **R14** | **R15** | **R16** | **R17** | **R18** |
| --- | --- | --- | --- | --- | --- | --- | --- | --- | --- | --- | --- | --- | --- | --- | --- | --- | --- | --- |
| C/ACT | >19 | >19 | >19 | >19 | >19 | >19 | >19 | 13-19 | 13-19 | 13-19 | 13-19 | 13-19 | 13-19 | 13-19 | 13-19 | <13 | <13 | <13 |
| Adherent | Yes | Yes | Yes | Yes | Yes | Yes | No | Yes | Yes | Yes | Yes | Yes | Yes | Yes | No | Yes | Yes | No |
| AA in last 6 mo | No | No | Yes | Yes | Yes | Yes |  | No | No | No | No | Yes | Yes | Yes |  |  |  |  |
| Step up in last 3 mo |  |  | No | No | No | Yes |  | No | No | No | Yes | No | Yes | Yes |  | No | Yes |  |
| SABA | <3d/w | ≥3d/w | <3d/w | ≥3d/w | ≥3d/w |  |  | <3d/w | ≥3d/w | ≥3d/w |  |  |  |  |  |  |  |  |
| Bud equiv |  |  |  | ≤400 | >400 |  |  |  | ≤400 | >400 |  |  | ≤400 | >400 |  |  |  |  |
| CONTROL ARM | No change  Path A | RCO^2^ | No change  Path A | Step up  Path B | No change  Path A | No change  Path A | No change  Path A | No change  Path C | Step up  Path B | No change  Path C | No change  Path C | Step up  Path B | Step up  Path B | No change  Path C | No change  Path C | Step up  Path B | RCO^3^ | No change  Path C |
| INTERVENTION ARM: Spirometry ratio equal to or above LLN (ie z score ≥ -1.64 “good”) | No change  Path A | RCO^2^ | No change  Path A | No change  Path A | No change  Path A | No change  Path A | No change  Path A | No change  Path C | No change  Path C | No change Path C | No change Path C | Step up  Path B | No change  Path C | No change  Path C | No change  Path C | Step up  Path B | RCO^3^ | No change  Path C |
| INTERVENTION ARM: Spirometry ratio below LLN (ie z score < -1.64 “bad”) | No change  Path A | Step up  Path B^1^ | No change  Path A | Step up  Path B^1^ | Step up  Path B^1^ | Step up  Path B^1^ | No change  Path A | Step up  Path B | Step up  Path B | Step up  Path B | Step up  Path B | Step up  Path B | Step up  Path B | Step up  Path B | No change  Path C | Step up  Path B | Step up  Path B | No change  Path C |

**Decision tree - path A – follow this path if there was a step down at the previous visit or following *R1/R3/R4/R5/R6/R7 – no change* at baseline**

| **Path A scenario** | **A1** | **A2** | **A3** | **A4** | **A5** | **A6** | **A7** | **A8** | **A9** | **A10** | **A11** | **A12** | **A13** | **A14** | **A15** | **A16** | **A17** | **A18** | **A19** | **A20** | **A21** | **A22** | **A23** | **A24** | **A25** | **A26** | **A27** |
| --- | --- | --- | --- | --- | --- | --- | --- | --- | --- | --- | --- | --- | --- | --- | --- | --- | --- | --- | --- | --- | --- | --- | --- | --- | --- | --- | --- |
| C/ACT | >19 | >19 | >19 | >19 | >19 | >19 |  |  | >19 |  |  | 13-19 | 13-19 | 13-19 |  |  | 13-19 | 13-19 | 13-19 | 13-19 | 13-19 | 13-19 | <13 |  |  |  | <13 |
| Adherent | Yes | Yes | Yes | Yes | Yes | Yes |  |  | No |  |  | Yes | Yes | Yes |  |  | Yes | Yes | Yes | Yes | Yes | No | Yes |  |  |  | No |
| AA in last 3 mo | No | No | Yes | Yes | Yes | Yes |  |  |  |  |  | No | No | No |  |  | Yes | Yes | Yes | Yes | Yes |  |  |  |  |  |  |
| Step up since last visit |  |  | No | No | No | Yes |  |  |  |  |  | Yes | No | No |  |  | Yes | Yes | No | No | No |  |  |  |  |  |  |
| SABA | <3d/w | ≥3d/w | <3d/w | ≥3d/w | ≥3d/w |  |  |  |  |  |  |  | <3d/w | ≥3d/w |  |  |  |  | <3d/w | <3d/w | ≥3d/w |  |  |  |  |  |  |
| Bud equiv |  |  |  | ≤400 | >400 |  |  |  |  |  |  |  |  |  |  |  | ≤400 | >400 | ≤400 | >400 |  |  |  |  |  |  |  |
| CONTROL ARM | Step down  Path A | RCO^2^ | No change  Path C | Step up  Path B | No change  Path C | No change  Path C |  |  | No change if adherent at previous visit  Path C |  |  | No change  Path C | No change  Path C | Step up  Path B |  |  | Step up  Path B | No change  Path C | Step up  Path B | No change  Path C | Step up  Path B | No change if adherent at previous visit  Path C | Step up  Path B |  |  |  | No change if adherent at previous visit  Path C |
|  |  |  |  |  |  |  |  |  | RCO if second consecutive episode of non-adherence^4^ |  |  |  |  |  |  |  |  |  |  |  |  | RCO if second consecutive episode of non-adherence^4^ |  |  |  |  | RCO if second consecutive episode of non-adherence^4^ |
| INTERVENTION ARM: Spirometry change score  >-1.6 AND <+1.6 (ie “good” / or stable spirometry) | Step down  Path A | RCO^2^ | No change  Path C | No change  Path C | No change  Path C | No change  Path C |  |  | No change if adherent at previous visit  Path C |  |  | No change  Path C | No change  Path C | No change  Path C |  |  | No change  Path C | No change  Path C | No change  Path C | No change  Path C | Step up  Path B | No change if adherent at previous visit  Path C | Step up  Path B |  |  |  | No change if adherent at previous visit  Path C |
|  |  |  |  |  |  |  |  |  | RCO if second consecutive episode of non-adherence^4^ |  |  |  |  |  |  |  |  |  |  |  |  | RCO if second consecutive episode of non-adherence^4^ |  |  |  |  | RCO if second consecutive episode of non-adherence^4^ |
| INTERVENTION ARM: Spirometry change score  ≤ -1.6 OR ≥+1.6  (ie “poor” / or less stable spirometry) | No change  Path C | Step up  Path B^1^ | No change  Path C | Step up  Path B^1^ | Step up  Path B^1^ | Step up  Path B^1^ |  |  | No change if adherent at previous visit  Path C |  |  | Step up  Path B | Step up  Path B | Step up  Path B |  |  | Step up  Path B | Step up  Path B | Step up  Path B | Step up  Path B | Step up  Path B | No change if adherent at previous visit  Path C | Step up  Path B |  |  |  | No change if adherent at previous visit  Path C |
|  |  |  |  |  |  |  |  |  | RCO if second consecutive episode of non-adherence^4^ |  |  |  |  |  |  |  |  |  |  |  |  | RCO if second consecutive episode of non-adherence^4^ |  |  |  |  | RCO if second consecutive episode of non-adherence^4^ |

**Decision tree - path B – follow this path if there was a step up at the previous visit**

| **Path B scenario** | **B1** | **B2** | **B3** | **B4** | **B5** | **B6** | **B7** | **B8** | **B9** | **B10** | **B11** | **B12** | **B13** | **B14** | **B15** | **B16** | **B17** | **B18** | **B19** | **B20** | **B21** | **B22** | **B23** | **B24** | **B25** | **B26** | **B27** |
| --- | --- | --- | --- | --- | --- | --- | --- | --- | --- | --- | --- | --- | --- | --- | --- | --- | --- | --- | --- | --- | --- | --- | --- | --- | --- | --- | --- |
| C/ACT |  |  |  |  |  |  |  |  | >19 | >19 | >19 | 13-19 | 13-19 |  | 13-19 | 13-19 | 13-19 | 13-19 | 13-19 | 13-19 | 13-19 | 13-19 |  |  |  | <13 |  |
| Adherent |  |  |  |  |  |  |  |  | No | Yes | Yes | Yes | yes |  | Yes | Yes | Yes | Yes | Yes | Yes | Yes | No |  |  |  |  |  |
| AA in last 3 mo |  |  |  |  |  |  |  |  |  |  |  | No | No |  | No | No | Yes | Yes | Yes | Yes | Yes |  |  |  |  |  |  |
| Step up since last visit |  |  |  |  |  |  |  |  |  |  |  | Yes | No |  | No | No | Yes | Yes | No | No | No |  |  |  |  |  |  |
| SABA |  |  |  |  |  |  |  |  |  | <3d/w | ≥3d/w |  | <3d/w |  | ≥3d/w | ≥3d/w |  |  | <3d/w | <3d/w | ≥3d/w |  |  |  |  |  |  |
| Bud equiv |  |  |  |  |  |  |  |  |  |  |  |  |  |  | ≤400 | >400 | ≤400 | >400 | ≤400 | >400 |  |  |  |  |  |  |  |
| CONTROL ARM |  |  |  |  |  |  |  |  | No change if adherent at previous visit  Path C | No change  Path C | RCO^2^ | No change  Path C | No change  Path C |  | Step up  Path B | No change  Path C | Step up  Path B | No change  Path C | Step up  Path B | No change  Path C | Step up  Path B | No change if adherent at previous visit  Path C |  |  |  | RCO^3^ |  |
|  |  |  |  |  |  |  |  |  | RCO if second consecutive episode of non-adherence^4^ |  |  |  |  |  |  |  |  |  |  |  |  | RCO if second consecutive episode of non-adherence^4^ |  |  |  |  |  |
| INTERVENTION ARM: Spirometry change score  >-1.6 AND <+1.6 (ie “good” / or stable spirometry) |  |  |  |  |  |  |  |  | No change if adherent at previous visit  Path C | No change  Path C | RCO^2^ | No change  Path C | No change  Path C |  | No change  Path C | No change  Path C | No change  Path C | No change  Path C | No change  Path C | No change  Path C | Step up  Path B | No change if adherent at previous visit  Path C |  |  |  | RCO^3^ |  |
|  |  |  |  |  |  |  |  |  | RCO if second consecutive episode of non-adherence^4^ |  |  |  |  |  |  |  |  |  |  |  |  | RCO if second consecutive episode of non-adherence^4^ |  |  |  |  |  |
| INTERVENTION ARM: Spirometry change score  ≤ -1.6 OR ≥+1.6  (ie “poor” / or less stable spirometry) |  |  |  |  |  |  |  |  | No change if adherent at previous visit  Path C | No change  Path C | Step up  Path B^1^ | Step up  Path B | Step up  Path B |  | Step up  Path B | Step up  Path B | Step up  Path B | Step up  Path B | Step up  Path B | Step up  Path B | Step up  Path B | No change if adherent at previous visit  Path C |  |  |  | RCO^3^ |  |
|  |  |  |  |  |  |  |  |  | RCO if second consecutive episode of non-adherence^4^ |  |  |  |  |  |  |  |  |  |  |  |  | RCO if second consecutive episode of non-adherence^4^ |  |  |  |  |  |

**Decision tree - path C – follow this path if there was no change at the previous visit (unless previous visit was baseline and the outcome was *R1/R3/R4/R5/R6/R7 – no change* at baseline; these will follow path A)**

| **Path C scenario** | **C1** | **C2** |  |  |  | **C6** | **C7** | **C8** | **C9** |  |  | **C12** | **C13** | **C14** | **C15** | **C16** | **C17** | **C18** | **C19** | **C20** | **C21** | **C22** |  | **C24** | **C25** |  | **C27** |
| --- | --- | --- | --- | --- | --- | --- | --- | --- | --- | --- | --- | --- | --- | --- | --- | --- | --- | --- | --- | --- | --- | --- | --- | --- | --- | --- | --- |
| C/ACT | >19 | >19 |  |  |  | >19 | >19 | >19 | >19 |  |  | 13-19 | 13-19 |  | 13-19 | 13-19 | 13-19 | 13-19 | 13-19 | 13-19 | 13-19 | 13-19 |  | <13 | <13 |  | <13 |
| Adherent | yes | yes |  |  |  | Yes | Yes | Yes | No |  |  | Yes | yes |  | Yes | Yes | Yes | Yes | Yes | Yes | Yes | No |  | Yes | Yes |  | No |
| AA in last 3 mo | No | No |  |  |  | Yes | Yes | Yes |  |  |  | No | No |  | No | No | Yes | Yes | Yes | Yes | Yes |  |  |  |  |  |  |
| Step up since last visit |  |  |  |  |  | Yes | No | No |  |  |  | Yes | No |  | No | No | Yes | Yes | No | No | No |  |  | No | Yes |  |  |
| SABA | <3d/w | ≥3d/w |  |  |  |  |  |  |  |  |  |  | <3d/w |  | ≥3d/w | ≥3d/w |  |  | <3d/w | <3d/w | ≥3d/w |  |  |  |  |  |  |
| Bud equiv |  |  |  |  |  |  | ≤400 | >400 |  |  |  |  |  |  | ≤400 | >400 | ≤400 | >400 | ≤400 | >400 |  |  |  |  |  |  |  |
| CONTROL ARM | If previous C/ACT>19  Step down  Path A | RCO^2^ |  |  |  | No change  Path C | Step up  Path B | No change  Path C | No change if adherent at previous visit  Path C |  |  | No change  Path C | No change  Path C |  | Step up  Path B | No change  Path C | Step up  Path B | No change  Path C | Step up  Path B | No change  Path C | Step up  Path B | No change if adherent at previous visit  Path C |  | Step up  Path B | RCO^3^ |  | No change if adherent at previous visit  Path C |
|  | If previous C/ACT≤19  No change  Path C |  |  |  |  |  |  |  | RCO if second consecutive episode of non-adherence^4^ |  |  |  |  |  |  |  |  |  |  |  |  | RCO if second consecutive episode of non-adherence^4^ |  |  |  |  | RCO if second consecutive episode of non-adherence^4^ |
| INTERVENTION ARM: Spirometry change score  >-1.6 AND <+1.6 (ie “good” / or stable spirometry) | Step down  Path A | RCO^2^ |  |  |  | No change  Path C | No change  Path C | No change  Path C | No change if adherent at previous visit  Path C |  |  | No change  Path C | No change  Path C |  | No change  Path C | No change  Path C | No change  Path C | No change  Path C | No change  Path C | No change  Path C | Step up  Path B | No change if adherent at previous visit  Path C |  | Step up  Path B | RCO^3^ |  | No change if adherent at previous visit  Path C |
|  |  |  |  |  |  |  |  |  | RCO if second consecutive episode of non-adherence^4^ |  |  |  |  |  |  |  |  |  |  |  |  | RCO if second consecutive episode of non-adherence^4^ |  |  |  |  | RCO if second consecutive episode of non-adherence^4^ |
| INTERVENTION ARM: Spirometry change score  ≤ -1.6 OR ≥+1.6  (ie “poor” / or less stable spirometry) | No change  Path C | Step up  Path B^1^ |  |  |  | Step up  Path B^1^ | Step up  Path B^1^ | Step up  Path B^1^ | No change if adherent at previous visit  Path C |  |  | Step up  Path B | Step up  Path B |  | Step up  Path B | Step up  Path B | Step up  Path B | Step up  Path B | Step up  Path B | Step up  Path B | Step up  Path B | No change if adherent at previous visit  Path C |  | Step up  Path B | RCO^3^ |  | No change if adherent at previous visit  Path C |
|  |  |  |  |  |  |  |  |  | RCO if second consecutive episode of non-adherence^4^ |  |  |  |  |  |  |  |  |  |  |  |  | RCO if second consecutive episode of non-adherence^4^ |  |  |  |  | RCO if second consecutive episode of non-adherence^4^ |

**Notes:**

***Overview of the algorithm (decision trees and treatment step table)***

The algorithm comprises the decision trees (above) and the treatment step table (Additional File 2). At the start of each visit, the child will land on a treatment step reflecting their current asthma preventer medication (see Additional File 2). Based on the factors included in the algorithm (C/ACT, adherence, asthma attack, step up, SABA, budesonide equivalent; and, in the intervention arm, spirometry; and at follow-up visits, what happened at the last visit), they will also be placed into one of the above scenarios and the decision tree will make a recommendation as to whether there should a step up, step down or no change to treatment or if the child should be referred for a clinical opinion. The treatment step table will then indicate where the child would step up or step down to.

If children are on a non-standard strength or frequency of inhaler, there is no treatment step for them to land on at the start of a visit. The centre will record the inhaler type and select “other strength or frequency of dose”. For these children, the algorithm will recommend RCO and will also make a recommendation in terms of step up, step down or no change. However (because there is no landing treatment step), the algorithm cannot make a recommendation as to the preventer medication that the child should take for the next three months.

***Application of the decision trees***

The location of the information required to apply the decision trees **at baseline** is recorded in the following sections of the case report forms

| C/ACT | Total score on Children’s Asthma Control Test or Children’s Asthma Control Test |
| --- | --- |
| Adherent | Adherence to ICS inhaler |
| AA in last 6 mo | Has there been an asthma attack in the last 6 months |
| Step up in last 3 mo | Has treatment been stepped up in the last 3 months |
| SABA | How frequently do they use their SABA inhaler |
| Bud equiv | What is the dose (as micrograms of budesonide equivalent) of their current ICS inhaler; based on current treatment (and for calculation of bud equivalent, refer to the treatment step table) |
| Spirometry | FEV1/FVC ratio in relation to Lower Level of Normal (LLN) |

The location of the information required to apply the decision trees in paths A, B and C (i.e. **at follow-up visits)** is recorded in the following sections of the case report forms

| C/ACT | Total score on Children’s Asthma Control Test or Children’s Asthma Control Test |
| --- | --- |
| Adherent | Adherence to ICS inhaler |
| AA in last 3 mo | Has there been an asthma attack in the last 3 months |
| Step up since last visit | Has treatment been stepped up in the last 3 months |
| SABA | How frequently do they use their SABA inhaler |
| Bud equiv | What is the dose (as micrograms of budesonide equivalent) of their current ICS inhaler (and for calculation of bud equivalent, refer to the treatment step table) |
| Spirometry | FEV1 change score in relation to +/-1.6 |

At any appointment, if the asthma inhaler technique is “not satisfactory after training” the child should be referred for clinical opinion. This rule should be applied before the other factors within the decision tree. The recommendation should NOT include a step up/step down/no change indication. Therefore, the recommendation from the algorithm should read: **Refer clinical opinion (RCO) – inhaler technique inadequate after training**.

At any appointment if the child has a separate long acting beta agonist (LABA) inhaler, the child should be referred for clinical opinion . This rule should be applied before the other factors within the decision tree. The recommendation should include RCO and a recommendation about step up/step down/no change, but will not recommend a specific treatment step. Therefore, the recommendation from the algorithm would read: Refer clinical opinion – step up; or refer clinical opinion – step down; or Refer clinical opinion – no change.

At any appointment, if the spirometry is more than ±4.5 z-scores, the algorithm will make a recommendation in terms of treatment, but also recommend “refer clinical opinion” since at such extreme values the change score calculation may lack accuracy, e.g. in young children no change in absolute FEV_1_ value may yield a change score of >1.6.

***Decision tree path at subsequent visits***

At each visit, for most scenarios on the decision trees, the recommendation includes the path that they will follow at the next visit. Please note that a child may be on a different path at each visit. *So, for example at baseline they may be recommended no change, path A; at three months they will follow Path A, but may end up with a recommendation at the end of the 3 month visit to Step up, Path B. At the 6 month visit, they will then follow Path B; and so on.*

- *If the decision tree makes a recommendation of step up, step down or no change and the treatment step table can provide the team with a preventer treatment recommendation* ***and*** ***the asthma team decide to follow the treatment recommendation*** *, the path indicated in the decision trees above will apply for the subsequent visit. There is also a question on the case report form that asks centres to confirm whether there has been a step up, step down or no change in preventer treatment.*
- *If the decision tree makes a recommendation of step up, step down or no change and the treatment step table can provide the team with an inhaler recommendation* ***and the asthma team decide not to follow the treatment recommendation****, the centres will confirm in the case report form whether there has been a step up, step down or no change. This will inform the path for the subsequent visit. If the clinical opinion was step down, the child will follow path A at the next visit. If the clinical opinion was step up, the child will follow path B at the next visit. If the clinical opinion was no change, the child will follow path C at the next visit.*
- *If the decision tree makes a recommendation of* ***refer*** ***clinical opinion*** *or the treatment step tables indicates* ***refer clinical opinion****, the asthma team will make a decision to step up, step down or make no change to treatment. The research team at centre will record this on the case report form. In these situations, the clinical opinion to step up, step down or make no change to treatment will determine the path for the next visit. If the clinical opinion was step down, the child will follow path A at the next visit. If the clinical opinion was step up, the child will follow path B at the next visit. If the clinical opinion was no change, the child will follow path C at the next visit.*

**Specific footnotes for the decision trees.**

^1^ These children (R2, R4, R5, R6, A2, A4, A5, A6, B11, C2, C6, C7, C8) should be flagged because only one step up during the trial in these scenarios is permitted. If they land on one of these scenarios for a second (or subsequent) time, the recommendation would be “No Change” and they would follow Path C at the next appointment.

2. Children who receive an RCO from the decision tree because they are well controlled/frequent SABA use. The recommendation should NOT include a step up/step down/no change indication. Therefore, the recommendation from the algorithm should read: **Refer Clinical Opinion (RCO) – well controlled asthma, but frequent use of SABA.**

3 Children who receive an RCO from the decision tree because of poor asthma control. The recommendation should NOT include a step up/step down/no change indication. Therefore, the recommendation from the treatment algorithm should read: **Refer Clinical Opinion (RCO) – poor asthma control.**

4 Children who receive an RCO from the decision tree because this is their second consecutive episode of non-adherence. The recommendation should NOT include a step up/step down/no change indication. Therefore, the recommendation from the treatment algorithm should read: **Refer Clinical Opinion (RCO) – second consecutive episode of poor adherence to treatment.**
