# Supplementary material for: Spirometry to manage asthma in children: study protocol for a randomised controlled trial (SPIROMAC)
Source: Trials. 2025 Sep 29;26:373. doi: 10.1186/s13063-025-09104-1 (PMC12482643; doi:10.1186/s13063-025-09104-1)
Supplement: Supplementary file 2 — Additional file 2. SPROMAC Algorithm recommended treatment step table. Algorithm treatment recommendations [file 13063_2025_9104_MOESM2_ESM.docx]

**Additional File 2 –Algorithm recommended treatment step table**

**TREATMENT RECOMMENDATIONS**

|  |  |  |  | **INTERVENTION ARM**:  At baseline: Intermediate/poor control (C/ACT <19) and Spirometry ratio below LLN ^4^.  At follow-up: Intermediate /poor control (C/ACT<19) and spirometry change score ≤-1.6 OR ≥+1.6 ^3^ | **STANDARD CARE ARM** (all scenarios)  **INTERVENTION ARM** (all scenarios apart from those indicated in previous column) | | | |
| --- | --- | --- | --- | --- | --- | --- | --- | --- |
| **Asthma medication (ICS/LABA/LTRA)^1^** | **Budesonide equivalent;  L (LABA)** | **BTS Step^2^** | **Drug no** | Age<12, Age ≥12 years | Age <12 years | | Age ≥12 years | |
|  |  |  |  | Step up^4^ | Step up^4^ | Step down^4^ | Step up^4^ | Step down^4^ |
| SABA only (no inhaled corticosteroid) | 0 | 1 | **0** |  | 2 | n/a | 2 | n/a |
| SABA only (no inhaled corticosteroid) plus LTRA | 0 | 2 | **1** |  | 4 | 0 | 4 | 0 |
| Beclomethasone (Clenil) 50 mcg MDI 2 puffs twice daily | 200 | 2 | **2** |  | 3 | 0 | 3 | 0 |
| Beclomethasone (Clenil) 100 mcg MDI 2 puffs twice daily | 400 | 2/3^5^ | **3** |  | 34 | 2 | 34 | 2 |
| Beclomethasone (Clenil) 50 mcg MDI 2 puffs twice daily plus LTRA | 200 | 3 | **4** |  | 5 | 2 | 5 | 2 |
| Beclomethasone (Clenil) 100 mcg MDI 2 puffs twice daily plus LTRA | 400 | 3 | **5** |  | 37 | 3 | 37 | 3 |
| Budesonide (Pulmicort Turbohaler) 100 mcg DPI 1 dose twice daily | 200 | 2 | **6** | 40 | 7 | 0 | 7 | 0 |
| Budesonide (Pulmicort Turbohaler) 200 mcg DPI 1 dose twice daily | 400 | 2/3^5^ | **7** |  | 41 | 6 | 41 | 6 |
| Budesonide (Pulmicort Turbohaler) 400 mcg DPI 1 dose twice daily | 800 | 3/4^6^ | **8** |  | 42 | 7 | 42 | 7 |
| Budesonide (Pulmicort Turbohaler) 100 mcg DPI 1 dose twice daily plus LTRA | 200 | 3 | **9** | 43 | 10 | 6 | 10 | 6 |
| Budesonide (Pulmicort Turbohaler) 200 mcg DPI 1 dose twice daily plus LTRA | 400 | 3 | **10** |  | 44 | 7 | 44 | 7 |
| Budesonide (Pulmicort Turbohaler) 400 mcg DPI 1 dose twice daily plus LTRA | 800 | 4 | **11** |  | 45 | 10 | 45 | 10 |
| Fluticasone (Flixotide Accuhaler) 50 mcg DPI 1 dose twice daily | 200 | 2 | **12** |  | 13 | 0 | 13 | 0 |
| Fluticasone (Flixotide Accuhaler) 100 mcg DPI 1 dose twice daily | 400 | 2/3^5^ | **13** |  | 28 | 12 | 28 | 12 |
| Fluticasone (Flixotide Accuhaler) 250 mcg DPI 1 dose twice daily | 1000 | 3/4^6^ | **14** |  | 29 | 13 | 29 | 13 |
| Fluticasone (Flixotide Accuhaler) 500 mcg DPI 1 dose twice daily | 2000 | 5 | **15** |  | A | A | 30 | 14 |
| Fluticasone (Flixotide Accuhaler) 50 mcg DPI 1 dose twice daily plus LTRA | 200 | 3 | **16** |  | 17 | 12 | 17 | 12 |
| Fluticasone (Flixotide Accuhaler) 100 mcg DPI 1 dose twice daily plus LTRA | 400 | 3 | **17** |  | 31 | 13 | 31 | 13 |
| Fluticasone (Flixotide Accuhaler) 250 mcg DPI 1 dose twice daily plus LTRA | 1000 | 4 | **18** |  | 32 | 17 | 32 | 17 |
| Fluticasone (Flixotide Accuhaler) 500 mcg DPI 1 dose twice daily plus LTRA | 2000 | 5 | **19** |  | A | A | 33 | 18 |
| Fluticasone (Flixotide Evohaler) 50 mcg MDI 1 puff twice daily | 200 | 2 | **20** |  | 21 | 0 | 21 | 0 |
| Fluticasone (Flixotide Evohaler) 50 mcg MDI 2 puffs twice daily | 400 | 2/3^5^ | **21** |  | 34 | 20 | 34 | 20 |
| Fluticasone (Flixotide Evohaler) 125 mcg MDI 2 puffs twice daily | 1000 | 3/4^6^ | **22** |  | 35 | 21 | 35 | 21 |
| Fluticasone (Flixotide Evohaler) 250 mcg MDI 2 puffs twice daily | 2000 | 5 | **23** |  | A | A | 36 | 22 |
| Fluticasone (Flixotide Evohaler) 50 mcg MDI 1 puff twice daily plus LTRA | 200 | 3 | **24** |  | 25 | 20 | 25 | 20 |
| Fluticasone (Flixotide Evohaler) 50 mcg MDI 2 puffs twice daily plus LTRA | 400 | 3 | **25** |  | 37 | 21 | 37 | 21 |
| Fluticasone (Flixotide Evohaler) 125 mcg MDI 2 puffs twice daily plus LTRA | 1000 | 4 | **26** |  | 38 | 25 | 38 | 25 |
| Fluticasone (Flixotide Evohaler) 250 mcg MDI 2 puffs twice daily plus LTRA | 2000 | 5 | **27** |  | A | A | 39 | 26 |
| Seretide Accuhaler 100 1 dose twice daily | 400 L | 3 | **28** |  | 31 | 13 | 31 | 13 |
| Seretide Accuhaler 250 1 dose twice daily | 1000 L | 4 | **29** |  | 32 | 28 | 32 | 28 |
| Seretide Accuhaler 500 1 dose twice daily | 2000 L | 5 | **30** |  | A | A | 33 | 29 |
| Seretide Accuhaler 100 1 dose twice daily plus LTRA | 400 L | 3 | **31** |  | 32 | 28 | 32 | 28 |
| Seretide Accuhaler 250 1 dose twice daily plus LTRA | 1000 L | 4 | **32** |  | 38 | 31 | 33 | 31 |
| Seretide Accuhaler 500 1 dose twice daily plus LTRA | 2000 L | 5 | **33** |  | A | A | 39 | 32 |
| Seretide Evohaler 50/25 MDI 2 puffs twice daily | 400 L | 3 | **34** |  | 37 | 21 | 37 | 21 |
| Seretide Evohaler 125/25 MDI 2 puffs twice daily | 1000 L | 4 | **35** |  | 38 | 34 | 38 | 34 |
| Seretide Evohaler 250/25 MDI 2 puffs twice daily | 2000 L | 5 | **36** |  | A | A | 39 | 35 |
| Seretide Evohaler 50/25 MDI 2 puffs twice daily plus LTRA | 400 L | 3 | **37** |  | 38 | 34 | 38 | 34 |
| Seretide Evohaler 125/25 MDI 2 puffs twice daily plus LTRA | 1000 L | 4 | **38** |  | RCO | 37 | 39 | 37 |
| Seretide Evohaler 250/25 MDI 2 puffs twice daily plus LTRA | 2000 L | 5 | **39** |  | A | A | RCO | 38 |
| Symbicort Turbohaler 100/6 I dose twice daily^7^ | 200 L | 3 | **40** |  | 41 | 6 | 41 | 6 |
| Symbicort Turbohaler 200/6 DPI 1 dose twice daily^7^ | 400 L | 3 | **41** |  | 44 | 7 | 44 | 7 |
| Symbicort Turbohaler 400/12 DPI 1 dose twice daily^7^ | 800 L | 4 | **42** |  | 45 | 41 | 45 | 41 |
| Symbicort Turbohaler 100/6 I dose twice daily plus LTRA^7^ | 200 L | 3 | **43** |  | 44 | 40 | 44 | 40 |
| Symbicort Turbohaler 200/6 DPI 1 dose twice daily plus LTRA^7^ | 400 L | 3 | **44** |  | 45 | 41 | 45 | 41 |
| Symbicort Turbohaler 400/12 DPI 1 dose twice daily plus LTRA^7^ | 800 L | 4 | **45** |  | 38 | 44 | 39 | 44 |
| Qvar 50 Autohaler or Aerosol MDI 1 puff twice a day | 200 | 2 | **47** |  | 48 | 0 | 48 | 0 |
| Qvar 100 Autohaler or Aerosol MDI 1 puff twice a day | 400 | 2/3^5^ | **48** |  | 34 | 47 | 34 | 47 |
| Qvar 100 Autohaler or Aerosol MDI 2 puffs twice a day | 800 | 3/4^6^ | **49** |  | 35 | 48 | 35 | 48 |
| Qvar 100 Autohaler or Aerosol MDI 4 puffs twice a day | 1600 | 5 | **50** |  | A | A | 36 | 49 |
| Qvar 50 Autohaler or Aerosol MDI 1 puff twice a day plus LTRA | 200 | 3 | **51** |  | 52 | 47 | 52 | 47 |
| Qvar 100 Autohaler or Aerosol MDI 1 puff twice a day plus LTRA | 400 | 3 | **52** |  | 37 | 48 | 37 | 48 |
| Qvar 100 Autohaler or Aerosol MDI 2 puffs twice a day plus LTRA | 800 | 4 | **53** |  | 38 | 52 | 38 | 52 |
| Qvar 100 Autohaler or Aerosol MDI 4 puffs twice a day plus LTRA | 1600 | 5 | **54** |  | A | A | 39 | 53 |
| Asmabec clickhaler 50 1 dose once daily | 100 | 2 | **55** |  | 56 | 0 | 56 | 0 |
| Asmabec clickhaler 50 1 dose twice daily | 200 | 2 | **56** |  | 57 | 55 | 57 | 55 |
| Asmabec clickhaler 100 1 dose twice daily | 400 | 2/3^5^ | **57** |  | 34 | 56 | 34 | 56 |
| Asmabec clickhaler 250 1 dose twice daily | 1000 | 3/4^6^ | **58** |  | 35 | 57 | 35 | 57 |
| Asmabec clickhaler 50 1 dose once daily plus LTRA | 100 | 3 | **59** |  | 60 | 55 | 60 | 55 |
| Asmabec clickhaler 50 1 dose twice daily plus LTRA | 200 | 3 | **60** |  | 61 | 56 | 61 | 56 |
| Asmabec clickhaler 100 1 dose twice daily plus LTRA | 400 | 3 | **61** |  | 37 | 57 | 37 | 57 |
| Asmabec clickhaler 250 1 dose twice daily plus LTRA | 1000 | 4 | **62** |  | 38 | 61 | 38 | 61 |
| Budelin Novolizer 200 1 dose once daily | 200 | 2 | **63** |  | 64 | 0 | 64 | 0 |
| Budelin Novolizer 200 1 dose twice daily | 400 | 2/3^5^ | **64** |  | 34 | 63 | 34 | 63 |
| Budelin Novolizer 200 2 doses twice daily | 800 | 3/4^6^ | **65** |  | 35 | 64 | 35 | 64 |
| Budelin Novolizer 400 1 dose once daily | 400 | 2/3^5^ | **66** |  | 34 | 63 | 34 | 63 |
| Budelin Novolizer 400 1 dose twice daily | 800 | 3/4^6^ | **67** |  | 35 | 66 | 35 | 66 |
| Budelin Novolizer 400 2 doses twice daily | 1600 | 5 | **68** |  | A | A | 36 | 67 |
| Budelin Novolizer 200 1 dose once daily plus LTRA | 200 | 3 | **69** |  | 70 | 63 | 70 | 63 |
| Budelin Novolizer 200 1 dose twice daily plus LTRA | 400 | 3 | **70** |  | 37 | 64 | 37 | 64 |
| Budelin Novolizer 200 2 doses twice daily plus LTRA | 800 | 4 | **71** |  | 38 | 70 | 38 | 70 |
| Budelin Novolizer 400 1 dose once daily plus LTRA | 400 | 3 | **72** |  | 37 | 66 | 37 | 66 |
| Budelin Novolizer 400 1 dose twice daily plus LTRA | 800 | 4 | **73** |  | 38 | 72 | 38 | 72 |
| Budelin Novolizer 400 2 doses twice daily plus LTRA | 1600 | 5 | **74** |  | A | A | 39 | 73 |
| Asmanex Twisthaler 200 mcg 1 dose once daily | 400 | 2/3^5^ | **75** |  | 34 | 2 | 34 | 2 |
| Asmanex Twisthaler 200 mcg 1 dose twice daily | 800 | 3/4^6^ | **76** |  | 35 | 75 | 35 | 75 |
| Asmanex Twisthaler 200 mcg 2 dose twice daily | 1600 | 5 | **77** |  | A | A | 36 | 76 |
| Asmanex Twisthaler 400 mcg 1 dose twice daily | 1600 | 5 | **78** |  | A | A | 36 | 76 |
| Asmanex Twisthaler 200 mcg 1 dose once daily plus LTRA | 400 | 3 | **79** |  | 37 | 75 | 37 | 75 |
| Asmanex Twisthaler 200 mcg 1 dose twice daily plus LTRA | 800 | 4 | **80** |  | 38 | 79 | 38 | 79 |
| Asmanex Twisthaler 200 mcg 2 dose twice daily plus LTRA | 1600 | 5 | **81** |  | A | A | 39 | 80 |
| Asmanex Twisthaler 400 mcg 1 dose twice daily plus LTRA | 1600 | 5 | **82** |  | A | A | 39 | 80 |
| Relvar Ellipta 92/22 one dose once a day | 1000 L | 4 | **83** |  | 85 | 34 | 85 | 34 |
| Relvar Ellipta 184/22 one dose once a day | 2000 L | 5 | **84** |  | A | A | 86 | 83 |
| Relvar Ellipta 92/22 one dose once a day plus LTRA | 1000 L | 4 | **85** |  | RCO | 37 | 86 | 37 |
| Relvar Ellipta 184/22 one dose once a day plus LTRA | 2000 L | 5 | **86** |  | A | A | RCO | 85 |
| Flutiform 50/5 mcg MDI inhaler or K-haler one puff twice daily | 200 L | 3 | **87** |  | 88 | 2 | 88 | 2 |
| Flutiform 50/5 mcg MDI inhaler or K-haler two puff twice daily | 400 L | 3 | **88** |  | 92 | 3 | 92 | 3 |
| Flutiform 125/5 mcg MDI inhaler or K-haler one puff twice daily | 500 L | 4 | **89** |  | 93 | 3 | 93 | 3 |
| Flutiform 125/5 mcg MDI inhaler or K-haler two puff twice daily | 1000 L | 4 | **90** |  | 94 | 89 | 94 | 89 |
| Flutiform 50/5 mcg MDI inhaler or K-halerone puff twice daily plus LTRA | 200 L | 3 | **91** |  | 92 | 87 | 92 | 87 |
| Flutiform 50/5 mcg MDI inhaler or K-haler two puff twice daily plus LTRA | 400 L | 3 | **92** |  | 94 | 88 | 94 | 88 |
| Flutiform 125/5 mcg MDI inhaler or K-haler one puff twice daily plus LTRA | 500 L | 4 | **93** |  | 94 | 89 | 94 | 89 |
| Flutiform 125/5 mcg MDI inhaler or K-haler two puff twice daily plus LTRA | 1000 L | 4 | **94** |  | RCO | 93 | 39 | 93 |
| Alvesco 80 mcg MDI one puff once daily | 160 | 2 | **95** |  | 96 | 0 | 96 | 0 |
| Alvesco 80 mcg MDI two puffs once daily | 320 | 2/3^5^ | **96** |  | 34 | 95 | 34 | 95 |
| Alvesco 160 mcg MDI one puff once daily | 320 | 2 | **97** |  | 34 | 95 | 34 | 95 |
| Alvesco 160 mcg MDI two puffs once daily | 640 | 3/4^6^ | **98** |  | 34 | 97 | 34 | 97 |
| Alvesco 160 mcg MDI two puffs twice daily | 1280 | 5 | **99** |  | A | A | 35 | 98 |
| Alvesco 80 mcg MDI one puff once daily plus LTRA | 160 | 3 | **100** |  | 101 | 95 | 101 | 95 |
| Alvesco 80 mcg MDI two puffs once daily plus LTRA | 320 | 3 | **101** |  | 37 | 96 | 37 | 96 |
| Alvesco 160 mcg MDI one puff once daily plus LTRA | 320 | 3 | **102** |  | 37 | 97 | 37 | 97 |
| Alvesco 160 mcg MDI two puffs once daily plus LTRA | 640 | 4 | **103** |  | 37 | 102 | 37 | 102 |
| Alvesco 160 mcg MDI two puffs twice daily plus LTRA | 1280 | 5 | **104** |  | A | A | 38 | 103 |

**Abbreviations:** mcg - microgram

A – this is a high dose of ICS for children aged <12 years; those who are on this medication at baseline or follow-up would receive the recommendation “refer for clinical opinion, child in on a high dose of budesonide equivalent for this age group.

n/a – children on SABA only cannot step down further; if the decision tree indicates step down; there will be no change to their treatment step (ie they will remain on SABA only until a step up is indicated by the decision tree)

RCO – refer for clinical opinion. If the decision tree indicates step up; the child will be referred to see the asthma team.

**Footnotes:**

^1^ The drugs included in the treatment step table are based on table 13 of the 2019 BTS guidelines. A number of the drugs listed in the guideline are not included on the algorithm because they are not licensed for use in children (eg Fostair). If new drugs/devices become available during the study period, these will be considered for inclusion on the algorithm if (i) they are licensed for children; and (ii) they are appropriate given the step up/down rules listed below.

Children who are taking a dose other than stated in the table are eligible for the study; and if there is not an appropriate landing step for them, the algorithm will return a “refer clinical opinion” outcome.

^2^ BTS step is based on the BTS 2019 guidelines^9^, and uses the following general rules:

|  | Age <12 years | Aged ≥12 years |
| --- | --- | --- |
| 100 to 200 bud equivalent no LABA or LTRA | Step 2 | Step 2 |
| 201 to 400 bud equivalent no LABA or LTRA | Step 3 | Step 2 |
| 401 to 1000 bud equivalent no LABA or LTRA^a^ | Step 4 | Step 3 |
| 1001 to 2000 bud equivalent no LABA or LTRA | Step 5 | Step 5 |
| 100 to 400 bud equivalent with LABA and/or LTRA | Step 3 | Step 3 |
| 401 to 1000 bud equivalent with LABA and/or LTRA | Step 4 | Step 4 |
| 1001 to 2000 bud equivalent with LABA and/or LTRA | Step 5 | Step 5 |

In the BTS 2019 guidelines 401-800 bud equivalent as monotherapy is not mentioned; on the basis of clinical experience, we are suggesting the classification in the table. Step 5 in the BTS 2019 guidelines states “refer patient for specialist care”, in practice the specialist will introduce ICS at doses of 801 to 2000 budesonide equivalent and this is how we have included in the above classification. If there are any children who join the study on SABA only (no ICS) but taking LTRA, these will be classified as BTS step 2. Children who are on SABA only at baseline will be classified as BTS step 1, we do not anticipate there will be many children on SABA only at baseline, so for the purposes of randomisation these children will be grouped with “other”.

^3^ In the intervention arm, for children with poor asthma control on low-dose ICS and reduced spirometry the algorithm will (where possible) recommend introducing LABA treatment rather than increasing the ICS to 400 mcg bud equivalent. This pertains to step up decisions in the INTERVENTION arm only (scenarios R8, R9, R10, R11, R12, R13, R14, R16, R17, A12, A13, A14, A17, A18, A19, A20, A21, A23 B12, B13, B15, B16, B17, B18, B19, B20, B21, C12, C13, C15, C16, C17, C18, C19, C20, C21, C24). The treatment recommendation only differs in respect to drug numbers 7 and 10. All other treatment recommendations will be identical to those shown in the final four columns of the above table.

^4^ General rules for step up/step down recommendations

| **Step up - children aged <12**   - Increase ICS to 400 mcg bud equivalent per day - Add LABA - Add LTRA - Increase ICS to 800/1000 mcg bud equivalent per day | **Step up - children aged** ≥**12**   - Increase ICS to 400 mcg bud equivalent per day - Add LABA - Add LTRA - Increase ICS to 2000 mcg bud equivalent per day | ***Step down (all ages)***   - Reduce ICS to 400 mcg bud equivalent per day - Remove LTRA - Remove LABA - Reduce ICS further |
| --- | --- | --- |
| When increasing/decreasing ICS, the recommended steps are: 200 bud equivalent per day; 400/500 bud equivalent per day; 800/1000 bud equivalent per day, 2000 bud equivalent per day. Children who step down from 200 bud equivalent per day will be on SABA only.  Where possible, delivery device should be maintained. In general, step up with the same inhaler device is recommended where possible. If the maximum dose is reached on a dry powder device and step up is still required, switching to MDI device is recommended. Therefore, when the maximum (for age) dose of ICS is reached on the Accuhaler device and both LABA and LTRA have been added, the recommendation is to change the delivery device to an Evohaler device (with spacer) at the same dose.  Children aged >=12 years who step up from Symbicort Turbohaler 400/12 DPI 1 dose twice daily (800 mcg bud equiv) will be recommended to step up to Seretide Evohaler 250/25 MDI/spacer 2 puffs twice daily (2000 mcg bud equiv). | | |

Additional rules for step up/step down recommendations

| - Flutiform 125/5 mcg MDI inhaler or K-haler one puff twice daily (budesonide equivalent 500L; drug 89); for step down treat as 400L so step down to 400 - Alvesco 80 mcg MDI two puffs once daily (budesonide equivalent 320; drug 96); for step up treat as 400 budesonide equivalent, so step up to 400L - Alvesco 160 mcg MDI one puff once daily (budesonide equivalent 320; drug 97); for step up treat as 400 budesonide equivalent, so step up to 400L - Alvesco 160 mcg MDI two puffs once daily (budesonide equivalent 640; drug 98); for step up treat as 400 budesonide equivalent, so step up to 400L - Alvesco 160 mcg MDI two puffs twice daily (budesonide equivalent 1280; drug 99); for step up treat as 1000 budesonide equivalent, so step up to 1000L |
| --- |

^5^ BTS step differs depending on age of child. For those aged >=12 years, BTS step is 2; for those aged <12 years, BTS step is 3.

^6^ BTS step differs depending on age of child. For those aged >=12 years, BTS step is 3; for those aged <12 years, BTS step is 4.

^7^ Symbicort Turbohaler is licensed for MART therapy (Maintenance And Reliever Therapy) in children 12 years and older. MART is sometimes referred to as SMART (Symbicort Maintenance And Reliever Therapy). The latest NICE guideline (BTS, NICE, SIGN; NG245, 2024)^10^ includes recommendation for low-dose MART therapy in children aged 5-11 (Symbicort used off-label). If children join the study on MART/SMART, the strength/frequency of the Symbicort Turbohaler will be recorded as “other” and the recommendation will be RCO. ART (As Required Treatment) also known as AIR (Anti-Inflammatory Reliever) is included as a recommendation for children aged 12 and over. If children join the study on ART/AIR, the strength/frequency of the Symbicort Turbohaler will be recorded as “other” and the recommendation will be RCO. We will capture if the child is on MART/SMART or ART/AIR^.^
